# The Mechanisms of Disease Caused by *Acinetobacter baumannii*

**DOI:** 10.3389/fmicb.2019.01601

**Published:** 2019-07-17

**Authors:** Faye C. Morris, Carina Dexter, Xenia Kostoulias, Muhammad Ikhtear Uddin, Anton Y. Peleg

**Affiliations:** ^1^Infection and Immunity Program, Department of Microbiology, Monash Biomedicine Discovery Institute, Monash University, Clayton, VIC, Australia; ^2^Department of Infectious Diseases, The Alfred Hospital and Central Clinical School, Monash University, Melbourne, VIC, Australia

**Keywords:** *A. baumannii*, host-pathogen interactions, animal models, immune response, bacterial virulence factors

## Abstract

*Acinetobacter baumannii* is a Gram negative opportunistic pathogen that has demonstrated a significant insurgence in the prevalence of infections over recent decades. With only a limited number of “traditional” virulence factors, the mechanisms underlying the success of this pathogen remain of great interest. Major advances have been made in the tools, reagents, and models to study *A. baumannii* pathogenesis, and this has resulted in a substantial increase in knowledge. This article provides a comprehensive review of the bacterial virulence factors, the host immune responses, and animal models applicable for the study of this important human pathogen. Collating the most recent evidence characterizing bacterial virulence factors, their cellular targets and genetic regulation, we have encompassed numerous aspects important to the success of this pathogen, including membrane proteins and cell surface adaptations promoting immune evasion, mechanisms for nutrient acquisition and community interactions. The role of innate and adaptive immune responses is reviewed and areas of paucity in our understanding are highlighted. Finally, with the vast expansion of available animal models over recent years, we have evaluated those suitable for use in the study of *Acinetobacter* disease, discussing their advantages and limitations.

## Introduction

*Acinetobacter baumannii* is a Gram negative, obligate aerobe, coccobacillus, and one of the most prevalent causes of nosocomial infections (Martín-Aspas et al., 2018). The burgeoning resistance of *A. baumannii* to primary antimicrobial therapies has created a deadly combination of pathogenicity and antimicrobial resistance that plagues hospitals (Roca et al., 2012). Classified as an ESKAPE pathogen (*Enterococcus faecium, Staphylococcus aureus, Klebsiella pneumoniae, Acinetobacter baumannii, Pseudomonas aeruginosa*, and *Enterobacter* species), carbapenem-resistant *A. baumannii* is considered the World Health Organization’s number one critical priority pathogen for which new therapeutics are urgently required (Shlaes and Bradford, 2018). Concerns continue to grow that without a significant intervention, hospital-acquired *A. baumannii* infections will soon be untreatable.

*Acinetobacter* phylogenetics has undergone significant changes, originally described as *Micrococcus*, with the designation of *Acinetobacter* only being proposed in the 1950’s (Peleg et al., 2008a). Since then, *Acinetobacter* taxonomy has been reclassified and over 50 different species have been identified to date (Harding et al., 2017a). While *A. baumannii*, *A. nosocomialis* and *A. pittii* are the most commonly isolated hospital species, *A. baumannii* international clonal types 1 and 2 are the most prominent, with lineage 3 largely restricted to Europe (Wallace et al., 2016; Weber et al., 2016). For more information on genomic diversity, the reader is directed to the following articles (Diancourt et al., 2010; Antunes et al., 2014; Touchon et al., 2014; Meumann et al., 2019).

**Figure 1:**
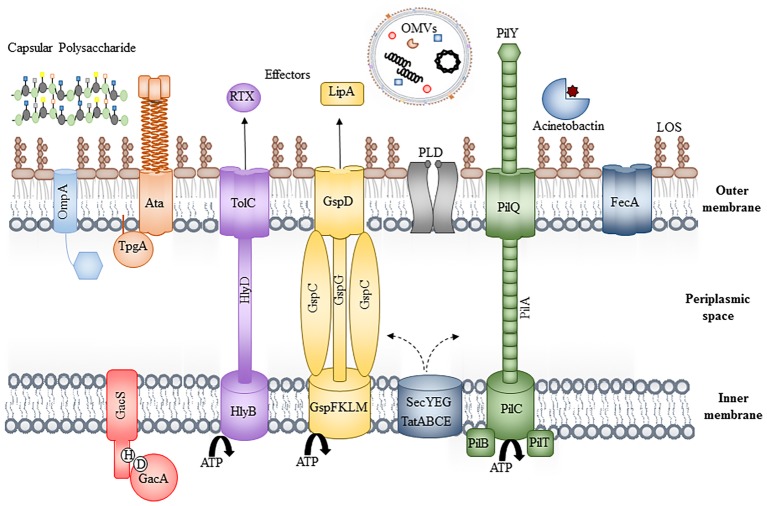
Bacterial virulence factors. Schematic of the bacterial cell envelope depicting some of the known virulence factors, including OmpA, the Type II, IV, and V secretion systems, phospholipase D (PLD), iron acquisition systems (Acinetobactin and FecA), the inner membrane two-component system, GacAS and extracellular factors, including lipid oligosaccharide, capsular polysaccharide, and outer membrane vesicles. For simplicity, peptidoglycan has been excluded and individual components are not to scale.

**Figure 2:**
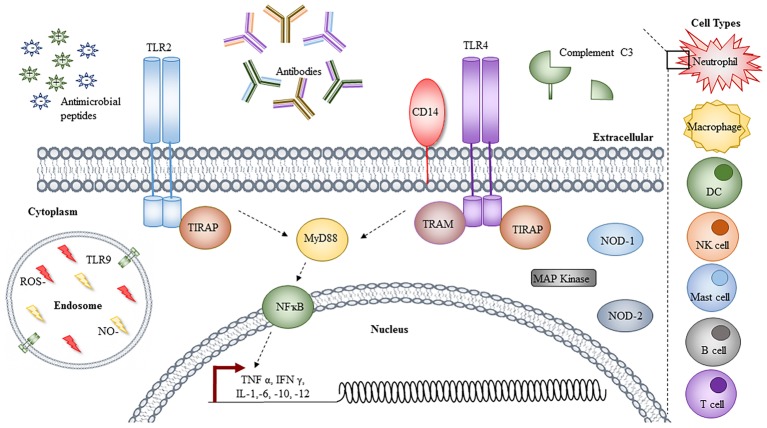
Immune responses to *Acinetobacter.* Immune cells involved in the clearance of *Acinetobacter* infections are denoted on the right-hand side, with an insert depicting the cellular components responsible. The toll-like receptor (TLR) 2 and 4 signaling leads to activation of NFĸB *via* MyD88, resulting in transcriptional activation and the synthesis of a range of cytokines and chemokines. Other cytoplasmic proteins shown to be involved in response to *Acinetobacter* infection are highlighted, with TLR9 localized to the endosome, in conjugation with reactive oxygen species (ROS) and nitric oxide (NO). Extracellular components, including cationic and anionic antimicrobial peptides, antibodies, and C3 complement are depicted left to right. For simplicity, not all proteins involved in the TLR signaling pathways are depicted and individual components are not to scale.

With only a limited number of “traditional” virulence factors, which are not always present or conserved across all strains, the mechanisms contributing to the success of *A. baumannii* are of increasing interest to researchers and clinicians alike. This article summarizes the knowledge of characterized virulence factors (depicted in Figure 1) and the host immune responses (depicted in Figure 2) that contribute to both its success and clearance *in vivo*, with a final overview of the available animal models, evaluating their advantages and limitations.

## Clinical Significance

*A. baumannii* causes a range of infections in both the hospital and community, including skin and soft tissue, urinary tract infections, meningitis, bacteremia, and pneumonia, with the latter being the most frequently reported infection in both settings (Dexter et al., 2015). Hospital-acquired infections are most commonly seen in critically ill patients; specific risk factors for developing an *A. baumannii* infection include prolonged hospital stays, immune suppression, advanced age, presence of comorbid diseases, major trauma or burns, previous antibiotic use, invasive procedures, and presence of indwelling catheters or mechanical ventilation (García-Garmendia et al., 2001; Robenshtok et al., 2006; Karageorgopoulos and Falagas, 2008; Wong et al., 2017). Due to the already poor prognosis of critically ill patients who acquire *A. baumannii* infections, it is difficult to attribute a definitive mortality rate (Freire et al., 2016); however crude morality rates have ranged from 23 to 68% (Eliopoulos et al., 2008).

Community-acquired infections present as a distinct and severe clinical syndrome in countries with hot and humid climates. These infections typically occur in individuals with underlying health conditions, including diabetes mellitus and chronic obstructive pulmonary disease, or in those that are heavy smokers or drink alcohol in excess (Falagas et al., 2007; Dexter et al., 2015). Mortality rates for community-acquired *A. baumannii* infections have been reported as high as 64% (Anstey et al., 1992; Patamatamkul et al., 2017); however, it is currently unknown as to whether host or bacterial factors are responsible for the difference in disease presentation between community and hospital infections.

## Bacterial Pathogenesis

### Virulence Factors

#### Outer Membrane Components

##### Outer Membrane Proteins

Outer membrane protein A (OmpA, previously Omp38) is the most abundant *A. baumannii* outer membrane protein (OMP) (Hee et al., 2008) and one of the most well-characterized virulence factors. OmpA is highly conserved, with 83 of 103 clinical isolates showing greater than 99% sequence identity, with the most diverse having 85% sequence identity (Ahmad et al., 2016; Ansari et al., 2018; Iyer et al., 2018). As such, OmpA has often been promoted as an attractive target for vaccine development. OmpA forms an eight-stranded β barrel in the OM, with a 2-nm pore diameter and C-terminal periplasmic globular extension, accommodating molecules up to 500 Da (Sugawara and Nikaido, 2012; Iyer et al., 2018). In contrast to other major porins, such as OmpF/C from *E. coli*, *A. baumannii* OmpA has significantly reduced permeability, thought to contribute to the overall reduction in *A. baumannii* OM permeability (Sugawara and Nikaido, 2012). To date, only the C-terminal periplasmic domain has been crystalized (1.6 Å) and shown to directly interact with peptidoglycan through Asp271 and Arg286 binding to diaminopimelate (Park et al., 2012). This interaction is thought to influence the packing of OMPs into outer membrane vesicles (OMVs), though this has yet to be confirmed, and may be merely a consequence of altered membrane homeostasis in an *ompA* mutant (Moon et al., 2012).

During normal growth and *in vivo* infection, OmpA is preferentially concentrated into OMVs (Moon et al., 2012). The interaction of OmpA (on the bacterial cell surface or OMVs) with eukaryotic cells induces cytotoxicity, through binding and adhesion to eukaryotic cell surface death receptors (Ahmad et al., 2016). Upon internalization, OmpA translocates to either the mitochondria or the nucleus (Hee et al., 2005, 2008; Rumbo et al., 2014). In the mitochondria, OmpA induces proapoptotic signals, through the activation of Bcl-2 family proteins, the release of cytochrome C and apoptosis-inducing factor (Hee et al., 2005). OmpA can also be translocated to the nucleus courtesy of its self-encoded nuclear localization signal (KTKEGRAMNRR), which is absent from other *A. baumannii* OMPs, and causes host DNA degradation in a DNase I-like manner (Hee et al., 2008; Choi et al., 2008a; Rumbo et al., 2014). In addition to its cytotoxic properties, OmpA modulates a range of other virulence attributes, including resistance to alternate complement-mediated killing through factor H binding and promoting adhesion to extracellular matrix proteins, including fibronectin, which is important for lung epithelial colonization (Kim et al., 2009; Smani et al., 2012).

Recent years have seen an expansion in our knowledge of other OMPs contributing to *A. baumannii* pathogenesis. Omp34 (otherwise termed Omp33–36) is highly conserved in *A. baumannii*, present in >1,600 strains with ≥98% identity (Rumbo et al., 2014). Similar to OmpA, Omp34 induces apoptosis in eukaryotic cells through caspase-dependent mechanisms and inhibition of autophagy, promoting bacterial persistence in the autophagosome (Rumbo et al., 2014; Jahangiri et al., 2018). Omp34 has also been shown to bind fibronectin and is selectively concentrated into OMVs (Smani et al., 2012). Shown to be important for systemic virulence in murine models, Omp34 is highly immunogenic, driving potent IgA/G/M antibody responses in sera from infected patients (Islam et al., 2011; Jahangiri et al., 2018). Additionally, OmpW also forms an eight-stranded OM β barrel protein, which is both highly immunogenic and concentrated in OMVs, though the functional role of this protein is thought to be related to iron acquisition and antibiotic resistance (Huang et al., 2015; Manuella et al., 2016).

Surface Antigen 1 (SurA) is another OMP that was identified from the chicken isolate CCGGD201101 (Liu et al., 2016). The relevance of this OMP to human clinical isolates is yet to be determined. While numerous other OMPs have been identified and characterized, these are discussed later in the context of bacterial nutrient acquisition, as they do not encode conventional virulence factors.

##### Lipopolysaccharide, Lipoolgiosaccharide, and Capsule

Similar to all other OM components, the Lipopolysaccharide (LPS), Lipoolgiosaccharide (LOS), and capsule are all synthesized in the cytoplasm and translocated to the outer leaflet of the cell envelop by dedicated proteinaceous machinery.

LPS is comprised of three distinct components: the lipid A anchor, glycosylated with core oligosaccharides, to which the O-antigen repeat is attached. In contrast, LOS does not contain O-antigen and instead has extended core oligosaccharides (Whitfield and Trent, 2014; Weber et al., 2016; Joseph and Stephen, 2018). Synthesized in the cytoplasm by the multistep Raetz pathway, both types are transported from the inner membrane to the cell surface by the Lpt pathway. Despite encoding two potential *waaL* O-antigen ligase homologs, *A. baumannii* does not produce O-antigen or LPS, and instead decorates the OM with LOS. Furthermore, subsequent analysis of these *waaL* genes has shown they are responsible for protein glycosylation (Iwashkiw et al., 2012).

Lipid A is the immunostimulatory component of LPS and LOS and previously thought to be essential in all Gram negative bacteria. Within the last decade, *Acinetobacter* has become only the third Gram negative pathogen capable of survival in the absence of lipid A, where previously only *Neisseria meningitidis* and *Moraxella catarrhalis* were thought to have this capacity (Moffatt et al., 2010). Lipid A minus *A. baumannii* was first identified in response to *in vitro* colistin exposure, resulting in inactivation of *lpxA*, *lpxC*, or *lpxD* in ATCC 19606 (Moffatt et al., 2010). Interestingly, with the exception of *lpxA*, *lpxC*, *lpxD*, and *lptD*, all other LOS biosynthetic genes are essential due to the resultant accumulation of toxic intermediates (Joseph and Stephen, 2018). For several years, the loss of lipid A was thought to be restricted to specific *A. baumannii* strains; however, recent efforts have shown that the levels of penicillin-binding protein PBP1a and, specifically, its glycosyltransferase activity are critical to the ability to lose lipid A (Boll et al., 2016). As lipid A is the major stimulus for toll like receptor (TLR) 4, it is unsurprising that lipid A minus strains reduce TLR4 signaling, but elevate TLR2 stimulation (Moffatt et al., 2013), thought to be as a result of the increased OM lipoprotein exposure. In contrast to LOS deficiency, *A. baumannii* more frequently modifies the lipid A moiety to promote antimicrobial resistance. Unlike other Gram negative pathogens, *A. baumannii* does not encode a PagP homolog; therefore, modification of the hexa-acylated lipid A occurs through the addition of one and two lauryl acyl chains during synthesis by the activity of LpxL and LpxM, respectively (Boll et al., 2015; Lopalco et al., 2017). This modification results in hepta-acylated lipid A that is more resistant to cationic antimicrobial peptides, less stimulatory of TLR4 and implicated in desiccation survival (Boll et al., 2015). By contrast to that of LOS minus strains, lipid A modification does not induce the same biological burden, and thus is readily detected in clinical isolates.

Capsule forms a protective layer on the extracellular surface, mediating resistance to cationic antimicrobial peptides and serum, subsequently enhancing *in vivo* survival (Geisinger and Isberg, 2015). Capsule loci in *A. baumannii* are highly variable, with conserved 5′ and 3′ genes capping the variable central cluster (Hu et al., 2013; Geisinger and Isberg, 2015). The 5′ *wza*, *wzb*, and *wzc* encode the assembly and export machinery complex spanning the inner and outer membranes, promoting transport of capsular polysaccaride from the periplasm to the cell surface (Kenyon and Hall, 2013; Senchenkova et al., 2015). The 3′ genes encode UDP-linked sugar synthases and epimerases, responsible for the conversion of UDP-D-glucose, UDP-D-galactose, and UDP-D-glucuronic acid to UDP-N acetyl-D-glucosaminuronic acid or UDP-N acetyl-D-galactosaminuronic acid, respectively (Hu et al., 2013; Kenyon and Hall, 2013). Other epimerases are encoded by central genes and/or at distinct sites around the chromosome, while glycosyltransferases and UndP lipid carriers responsible for the construction of the repeating unit, work in concert with wzx and wzy to translocate these components to the periplasm for polymerization and presentation to the Wzabc complex (Kenyon and Hall, 2013).

Capsule production is negatively regulated by the BfmRS two-component regulatory system (TCS) in response to environmental stimuli, including particular antibiotics (chloramphenicol and erythromycin), resulting in increased expression and antimicrobial resistance (Geisinger and Isberg, 2015). Furthermore, the presence or absence of capsule has also been linked to *Acinetobacter* phenotypic switching, whereby the opaque virulent form is characterized by enhanced capsule production, in contrast with the translucent avirulent form, which displays two-fold less capsule production (Chin et al., 2018).

##### Phospholipase

Phospholipases are well-established virulence factors and *Acinetobacter* encodes both phospholipase C and D enzymes, differentiated by their cleavage position preference resulting in a phospho head group (phospholipase C) or phosphatidic acid and a separate head group (phospholipase D) (Schmiel and Miller, 1999). *A. baumannii* encodes two phospholipase C and three phospholipase D enzymes, all with substrate specificity toward the eukaryotic membrane component, phosphatidylcholine (Stahl et al., 2015; Fiester et al., 2016). Interestingly, both enzymes are transcriptionally regulated by the ferric uptake regulator (Fur) and display hemolytic activity against human erythrocytes, aiding in iron acquisition (Fiester et al., 2016). Consistent with this important role, phospholipase C is conserved across numerous strains, including ATCC 19606, ATCC 17978, ACICU, AYE, and AB0057 (Fiester et al., 2016).

Similarly, the three phospholipase D genes are associated with serum resistance, epithelial cell invasion, and *in vivo* pathogenesis (Jacobs et al., 2010; Stahl et al., 2015). Two phospholipase D enzymes appear to be as a result of a gene duplication, identifiable by two catalytic domains containing the HxKx4Dx6GS/GGxN (HKD) motif, while phospholipase D3 contains only one (Stahl et al., 2015). Despite the similarity, phospholipase D2 has been shown to be more important for invasion than the other two, though the deletion of all three induces only marginal defects in virulence in a *Galleria mellonella* model (Stahl et al., 2015).

#### Secretion Systems

*Acinetobacter* encodes a diverse range of secretion systems. The Type I secretion system (T1SS) is a tripartite system, delivering proteins from the cytosol to the extracellular environment. In *Acinetobacter*, the T1SS is homologous to that of the prototypical HlyBD-TolC system from *E. coli*, consisting of an IM ATP-binding protein, periplasmic adaptor, and OM pore (Harding et al., 2017b). *A. nosocomialis* strain M2 was the first *Acinetobacter* strain shown to encode an active T1SS, with two putative effector proteins, RTX (containing an RTX toxin domain) and Bap (homologous to the biofilm associated protein) (Harding et al., 2017b). Interestingly, the activity of the T1SS in this strain was shown to have a direct impact on the Type VI secretion system (T6SS), suggestive of cross talk between these systems (Harding et al., 2017b).

The Type IV secretion system (T4SS) is responsible for conjugative transfer of DNA, plasmids, and other mobile genetic elements. To date, only three reports address the T4SS in *Acinetobacter* (Smith et al., 2007; Iacono et al., 2008; Liu et al., 2014). *A. baumannii* strain ATCC 17978 encodes eight genes homologous to those of the *Legionella/Coxiella* T4SS, while strains ACICU and TYTH-1 harbor plasmids encoding complete *tra* loci on pACICU2 and pAB-CC, respectively (Smith et al., 2007; Iacono et al., 2008; Liu et al., 2014). While these features are of critical importance in the transfer of genetic material, particularly that of antibiotic resistance determinants, their role in host-pathogen interactions has yet to be elucidated.

T6SSs are capable of targeting both eukaryotic and prokaryotic cells, though in *Acinetobacter* the T6SS exclusively targets other bacteria, secreting a range of toxins, including peptidoglycan hydrolases, nucleases, or those targeting cell membranes (Elhosseiny and Attia, 2018; Fitzsimons et al., 2018). Interestingly, despite its role in bacterial competition, clinical isolates with active T6SS are isolated from immunocompromised patients at higher frequencies (Repizo, 2017), suggestive of a competitive advantage, although this may be due to their bactericidal activity against competing pathogens.

##### Type II Secretion System

The Type II secretion system (T2SS) is a two step secretion mechanism, dependent on Sec or Tat for substrate translocation to the periplasm prior to secretion. The T2SS was first described in ATCC 17978, with the apparatus encoded by genes designated, general secretory pathway (GspA-O), distributed across discrete clusters and not a single operon (Eijkelkamp et al., 2014). Effector proteins include enzymes such as lipase, elastase, alkaline phosphatase, and phospholipases, critical for *A. baumannii* virulence (Elhosseiny and Attia, 2018).

In *A. baumannii*, specific T2SS effectors include the lipases, LipA and LipH, hydrolyzing long-chain fatty acids as carbon sources for growth, the metallo-endopeptidase, CpaA, responsible for fibrinogen and factor V degradation, while the PilD peptidase is shared between the T2SS and T4SS (Harding et al., 2016; Johnson et al., 2016; Weber et al., 2017). The importance of this system is delineated by the observed attenuation of various mutants (*gspD* and *lipA*) in both *G. mellonella* and murine models (Harding et al., 2016; Johnson et al., 2016).

##### Type V Secretion System

The Type V secretion system (T5SS) (autotransporters) is the simplest and most widespread secretion system in Gram negative bacteria (Henderson et al., 2000). They are identifiable by their distinct domain architecture, including a N-terminal Sec-dependent signal peptide, a central passenger domain and C-terminal β barrel (Henderson et al., 2004). To date, five subdivisions of this family have been identified termed Type Va-Ve (Leo et al., 2012); however, *Acinetobacter* encodes only two, Type Vb (AbFhaB/C and CdiA/B) and one Type Vc (Ata) (Bentancor et al., 2012a,b; Pérez et al., 2017; Elhosseiny and Attia, 2018).

In the Type Vb subclass, the passenger and β domains are encoded as two distinct proteins from an operon termed TspA (AbFhaB) and TspB (AbFhaC), respectively (Jacob-Dubuisson et al., 2001; Pérez et al., 2017). AbFhaC encodes the 16-stranded β barrel, with two periplasmic polypeptide transport-associated domains for the recognition and translocation of AbFhaB to the cell surface, where the arginine-glycine-aspartate (RGD) motif binds eukaryotic integrin and fibronectin molecules (Jacob-Dubuisson et al., 2009; Pérez et al., 2017). Interestingly, while disruption of AbFhaC and subsequent loss of AbFhaB result in increased fertility of *Caenorhabditis elegans* and increased murine survival, it does not completely attenuate virulence (Pérez et al., 2017).

Contact-dependent inhibition (CDI) was first identified in 2005 as a T6SS-independent mechanism of bacterial cell killing. CdiA/B are considered to be Type Vb autotransporters, whereby CdiB forms the OM pore for the secretion of the CdiA toxin (containing numerous filamentous hemagglutinin domains) (Harding et al., 2017b). In contrast to traditional Type Vb autotransporters, a third component termed CdiI encodes an immunity protein (Harding et al., 2017b). Initially identified in *A. nosocomialis*, this operon has since been identified in the *A. baumannii* strains ATCC 19606 and 1225 (Harding et al., 2017b), although the mechanism of killing has yet to be determined.

Type Vc, Ata forms a trimeric autotransporter, with an extended signal peptide, and a smaller C-terminal β domain. Each monomer encodes 101 amino acids, contributing four β strands to the final homotrimer (Bentancor et al., 2012a). The passenger domain of each monomer consists of three subdomains, head, neck, and stalk, which trimerise to form the functional moiety (Koiwai et al., 2016). Ata contains four pentameric collagen-binding consensus sequences (SVAIG) and an RGD motif important for binding to extracellular matrix and basal proteins, including collagen I, III, IV, and V and laminin (Bentancor et al., 2012a). Deletion of this protein significantly reduces the ability of ATCC 17978 to form biofilms and completely attenuates *in vivo* virulence (Bentancor et al., 2012a). Interestingly, Ata is produced in concert with TpgA, an OM lipoprotein anchor for Ata and although similar chaperones have been observed for other trimeric autotransporters, TpgA is unique in its OM localization (Ishikawa et al., 2016).

##### Efflux Systems

Bacterial efflux systems are membrane spanning, tripartite systems exhibiting broad substrate specificity, extruding potentially toxic compounds from the periplasm to the extracellular environment. To date, six bacterial efflux pump families have been identified, including the major facilitator superfamily (MFS), the multidrug and toxin extrusion family (MATE), the small multidrug resistance family (SMR), the resistance-nodulation-cell division superfamily (RND), and the proteobacterial antimicrobial compound efflux family (Du et al., 2018). In other Gram negative pathogens, efflux pumps play critical roles in the extrusion of bile salts and antimicrobial fatty acids and peptides or actively secrete virulence factors such as siderophores (Du et al., 2018). In *Acinetobacter*, AceI and the AdeABC efflux pumps promote resistance to biocides and aminoglycosides, respectively (Marchand et al., 2004; Hassan et al., 2013; Liu et al., 2018). While the majority of characterized efflux pumps have only been linked to the exclusion of toxic molecules, the AdeABC pump has been shown to impact bacterial fitness *in vivo* (Yoon et al., 2016). Overexpression of this particular pump in the drug susceptible strain BM4587 was shown to reduce bacterial burdens in the lungs and spleen at 8 h post infection when administered *via* the intraperitoneal route (Yoon et al., 2016). Conversely, transposon insertion mutants in AdeIJK and the toluene tolerance efflux transporter in AB5075 and ATCC17978, respectively, result in reduced bacterial persistence in *Galleria* larvae and a murine pneumonia model (Wang et al., 2014; Gebhardt et al., 2015). Although the mechanisms by which these efflux pumps contribute to virulence have yet to be elucidated, their complex genetic regulation implies a more significant role in bacterial homeostasis that has yet to be determined.

##### Outer Membrane Vesicles

OMVs are small, spherical vesicles, ranging in size between 10 and 300 nm, produced by all Gram negative species examined to date under varying growth conditions, indicative of an evolutionary conserved mechanism (Roier et al., 2016). In contrast to the name, these vesicles are an encapsulation of cytoplasmic components, IM, periplasmic proteins, and OM (Kulkarni and Jagannadham, 2014; Roier et al., 2016). Despite previously being considered a consequence of cell envelope stress, vesicle formation has recently been shown to be a natural process, though the exact mechanism of their biogenesis and differential packaging regarding the enrichment or depletion of particular OMPs or lipid species has yet to be determined (McBroom and Kuehn, 2005, 2007; Roier et al., 2016). It is unclear whether a dedicated mechanism exists or whether this is just a general secretory pathway responsive to different environmental conditions (Kato et al., 2002; Kuehn and Kesty, 2005; McMahon et al., 2012; Cahill et al., 2015). Currently, three hypotheses exist for their biogenesis: firstly, the loss of OM lipoprotein-peptidoglycan interactions leads to membrane protrusion and vesicle formation; second, the accumulation of misfolded proteins and peptidoglycan fragments in the periplasm lead to membrane bulging; third, the enrichment of molecules driving membrane curvature induces vesicle formation (Roier et al., 2016). Roiser et al. have shown that the OM lipoprotein VacJ in *Haemophilus influenzae* and *Vibrio cholerae* influences OMV phospholipid content, while in *A. baumannii* OmpA influences the OMP composition and abundance in OMVs, though further investigation is required to elucidate the mechanism (Moon et al., 2012; Roier et al., 2016).

OMVs have long been hypothesized to function as a bacterial defense mechanism against the host innate immune system (Beceiro et al., 2013) due to their association with bacterial signaling, modulation of host-pathogen interactions and immune evasion. *A. baumannii* OMVs provide an important mechanism for the secretion of OmpA and other putative virulence factors to host cells, through interactions with eukaryotic cholesterol micro-domains (Kwon et al., 2009; Jin et al., 2011). *In vivo* OMVs can stimulate immune responses through the activation of TLR signaling or modulate immune evasion through sequestration of innate immune factors (Kaparakis-Liaskos and Ferrero, 2015). For example in *E. coli*, OMVs have been shown to be produced in response to treatment with antimicrobial peptides, while in *H. influenzae* and *Moraxella catarrhalis*, OMVs promote serum resistance through the depletion of complement factors (Thuan Tong et al., 2007; Manning and Kuehn, 2011; Roier et al., 2016). Furthermore, OMVs provide a unique opportunity for bacterial dissemination of encapsulated genetic material within communities or across species. *Pseudomonas aeruginosa* OMVs have been shown to contain DNA and RNA capable of modulating host immune responses (Koeppen et al., 2016; Bitto et al., 2017). Similarly, multiple groups have shown *A. baumannii* clinical isolates utilize OMVs as a mechanism for dissemination of plasmids containing carbapenem resistance genes, including *bla*_OXA-24_ and *bla*_NDM-1_ (Rumbo et al., 2011; Chatterjee et al., 2017), highlighting the versatility of this system and the requirement for more in-depth analysis.

### Nutrient Acquisition

#### Metal Acquisition

##### Iron

Iron is the most restricted nutrient in the human body. Bacteria have developed two scavenging mechanisms: direct uptake through receptors and transporters and high-affinity secreted iron chelator proteins (siderophores) (Eijkelkamp et al., 2011; De Léséleuc et al., 2014). *A. baumannii* strains encode up to five different siderophores. Cluster 1 comprises eight genes and a MFS family efflux pump, cluster 2 (only present in ATCC 17978) includes 15 genes with a separate MFS and MATE efflux pumps, cluster 3 is the well-characterized acinetobactin and ABC efflux pump, while cluster 4 is only found in strain 8399, and cluster 5 is present in most strains with the exception of ATCC 17978 (Eijkelkamp et al., 2011). Despite cluster variability, those described to date are all transcriptionally regulated by Fur, identifiable by the presence of a Fur box sequence (a palindromic 25-nucleotide sequence) in their respective promoters (Eijkelkamp et al., 2011). In isolates, such as LAC-4 and SDF, the iron transporters and receptors FecI, FecR, and FecA are reported to enhance pathogenesis and growth capabilities of these strains through the utilization of heme (De Léséleuc et al., 2014).

Given the importance of iron-scavenging systems, it is unsurprising that disruption of these mechanisms causes dysregulation of other systems and virulence attenuation. In ATCC 17978, iron limitation results in a downregulation of Type 1 and Type IV pili (Eijkelkamp et al., 2011), while in ATCC 19606, disruption of the acinetobactin OM receptor, BauA, or the biosynthetic component, BasD, impairs intracellular persistence and killing of lung epithelial cells *in vitro*, though only varying degrees of attenuation are observed in *G. mellonella* and murine models (Gaddy et al., 2012). Similarly, disruption of Fur, iron-sulfur cluster assembly system (Isc), and/or other putative iron transporters (FeoA), leads to reduced bacterial biofilm formation, oxidative stress resistance, and *in vivo* pathogenesis (Ajiboye et al., 2018; Álvarez-Fraga et al., 2018). The phenotypes reported for *fur* mutants should be judged with caution due to the cross regulation with OxyR and SoxRS systems, which are associated with reactive oxygen detoxification and superoxide dismutase activation, and result in increased intracellular iron interacting with free radicals (Ajiboye et al., 2018). In contrast to that observed in the *G. mellonella* model, Fleming et al. reported that *A. baumannii* pathogenesis can be alleviated by the supply of excess iron to the surrounding environment when using a murine wound infection model, preventing the activation of iron-scavenging systems and virulence (Fleming et al., 2017).

##### Zinc and Manganese

Similar to iron, zinc is biologically important, acting as both a natural catalyst and cofactor for numerous proteins, with as many as 8% of *E. coli-*encoded proteins containing zinc-binding domains (Hood and Skaar, 2012). During the course of infection, the host can sequester metals by the action of the chelator protein calprotectin in a tactic termed “nutritional immunity” (Hood and Skaar, 2012; Nairn et al., 2016). *A. baumannii* regulates intracellular zinc concentrations by the activity of the ZnuABC transporter and ZigA GTPase, transcriptionally controlled by Zur (Mortensen et al., 2014; Nairn et al., 2016). While ZnuABC imports zinc into the cell, ZigA promotes its release through HutH activation and histidine catabolism (Mortensen et al., 2014; Nairn et al., 2016). This delicate balance between availability and toxicity results in a mild attenuation of *zigA* mutants in a murine pneumonia model, with less systemic dissemination from the lungs after infection (Nairn et al., 2016).

Although not characterized to the same degree, manganese starvation is of equal importance, whereby the NRAMP family transporter MumT and urea carboxylase, MumC, facilitate manganese accumulation in response to calprotectin (Juttukonda et al., 2016). Disruption of *mumT* significantly impacts bacterial colonization and dissemination from the lungs during pneumonia, a phenotype that is abolished in calprotectin-deficient mice (Juttukonda et al., 2016), further emphasizing the interplay of host and bacterial proteins in pathogenesis.

### Community Interactions

#### Quorum Sensing

The ability of bacteria to sense, respond to, and communicate with neighboring cells is critical to the success of the population. Quorum sensing is the process by which bacteria detect and respond to hormone like molecules, such as acyl homoserine lactone, regulating numerous phenotypes, including motility and biofilm formation (Bhargava et al., 2010; Rutherford and Bassler, 2012). In the LuxRI family of proteins, LuxI synthesizes the acyl homoserine lactone, which interacts with the LuxR protein mediating the transcriptional response (Bhargava et al., 2010). In *A. baumannii*, *abaI* is responsible for the synthesis of N-(3-hydroxydodecanoyl)-L-HSL (3-hydroxy-C12-HSL), functioning in conjugation with the *abaR* LuxR homolog, to regulate biofilm maturation (Niu et al., 2008). Furthermore, the importance of this system in virulence has been demonstrated by the significant increase in murine survival rates during infection with *abaI* mutants (Bhuiyan et al., 2016).

#### Biofilm Formation

Biofilms are a bacterial lifestyle, constituting dynamic community environments, comprised of a heterogeneous protein matrix, nucleic acids, polysaccharides, and bacterial microcolonies, dispersed with water channels (Hall-Stoodley et al., 2004). *A. baumannii* forms biofilms on both biotic and abiotic surfaces, promoting survival on indwelling medical devices, hospital surfaces, or in otherwise unfavorable conditions. Biofilm formation is a multistage process, commencing with the initial attachment, proceeding to strong adhesion and aggregation of cells into microcolonies, followed by biofilm growth and maturation, prior to cell dispersal into the environment (Hall-Stoodley et al., 2004).

Biofilm formation on abiotic surfaces has contributed to the success of this pathogen in hospital environments, with their ability to adhere to medically relevant surfaces, such as titanium and polystyrene (Loehfelm et al., 2008; Brossard and Campagnari, 2011). Numerous strains encode for the Csu Type 1 chaperone-usher pili, associated with cell-to-cell attachment and biofilm initiation (Tomaras et al., 2008; Brossard and Campagnari, 2011). The operon contains six genes *csuA/B*, *A*, *B*, *C*, *D*, and *E*, encoding for pili and minor subunits, the chaperone, the usher and adhesive tip, respectively (Moon et al., 2017). Despite being predominantly regulated by BfmRS TCS, the GacAS TCS also modulates its expression, in addition to a putative folate-responsive riboswitch identified in the *csu* and *bfmR* promoters (Cerqueira et al., 2014; Moon et al., 2017), emphasizing the interplay between environmental signals and population dynamics. Despite BfmRS being predominantly associated with abiotic biofilms, this system is also linked to eukaryotic cell adhesion and antimicrobial resistance (Liou et al., 2014).

*A. baumannii* also produce the T1SS effector biofilm-associated protein, Bap, associated with abiotic and biotic biofilms. Homologous to that previously identified in *Staphylococcus*, it is defined by its immense size (over 800 kDa) and immunoglobulin-like fold (Loehfelm et al., 2008; Brossard and Campagnari, 2011). In contrast to *Staphylococcus*, *A. baumannii* Bap contains four modules, A–D, with numerous internal repeats, promoting cell-to-cell adhesion and eukaryotic cell adhesion, through modulation of cell surface hydrophobicity (Loehfelm et al., 2008; Brossard and Campagnari, 2011). Interestingly, despite their clearly defined roles in biofilm formation, numerous strains contain mutations which disrupt the *csu* operon or *bap* gene abolishing their production, though the significance of these findings has yet to be determined (Goh et al., 2013; Wright et al., 2016).

In addition to the Csu pilus, *A. baumannii* encodes a second shorter (29 nm in length) chaperone usher Pap pilus, homologous to that of *E. coli* P pilin that is associated with eukaryotic cell adhesion (De Breij et al., 2009; Marti et al., 2011; Cerqueira et al., 2014). In addition to pilus complexes, proteins such as the autotransporter Ata and the extracellular poly-β-1,6-*N*-acetylglucosamine (PNAG) encoded for by *pgaABCD* locus have also been shown to mediate attachment and adhesion of cells in the biofilm (Choi et al., 2009; Brossard and Campagnari, 2011).

### Genetic Regulation of Virulence Phenotypes

#### Transcriptional Regulation

In addition to TCS, a recent study identified a Tet-R family transcription factor (encoded by *ABUW_1645* in strain AB5075) involved in the regulation of phenotypic switching between two colony types, termed opaque and translucent (Tipton et al., 2015; Chin et al., 2018). These morphological differences were not due to genetic mutations, and instead were driven by changes in the expression of *ABUW_1645*, which regulates ~70% of the differentially expressed genes including capsule biosynthesis (Chin et al., 2018). Interestingly, it is this phase variation and morphological difference that causes striking differences in *in vivo* pathogenesis, with the translucent form being avirulent, and frequent reversion to the opaque form is observed in cultures recovered from murine lungs post infection (Tipton et al., 2015; Chin et al., 2018). Consistent with this virulence phenotype, the opaque form shows resistance to cathelicidin-related antimicrobial peptides, reactive oxygen species (ROS), lysozyme, disinfectant, and desiccation. Thus, unsurprisingly, the opaque form is routinely isolated from clinical samples (Chin et al., 2018). The translucent form is thought to be associated with environmental settings and bacteriophage resistance, with its increased biofilm formation at lower temperatures, reduced capsule and upregulated nutrient acquisition and catabolism-related genes (Chin et al., 2018).

##### H-NS

The H-NS transcription factor is associated with silencing horizontally acquired and/or AT-rich genes, limiting their potentially detrimental effects (Eijkelkamp et al., 2013). Previous studies have shown disruption of *hns* in either ATCC 17978 or clinical isolates results in a myriad of phenotypes including hypermotility, increased colistin resistance, adhesion to epithelial cells, and *in vivo* virulence in *Caenorhabditis elegans* (Eijkelkamp et al., 2013; Deveson Lucas et al., 2018). Transcriptomic analysis of *hns* disruption mutants reveals a vast number of dysregulated genes, including the significant overexpression of those associated with quorum sensing, OMPs, T5SS, T6SS, and fatty acid biosynthesis (Eijkelkamp et al., 2013).

##### Two-Component Regulatory Systems

Two-component regulatory systems (TCSs) impact a diverse range of phenotypes by modulating transcriptional regulation. They are usually found as a pair of regulatory proteins, including a membrane-bound sensor kinase and a separate DNA-binding transcriptional regulator, which respond to environmental conditions and/or cell stress (Stock et al., 2000; Zschiedrich et al., 2016). To our knowledge, six TCSs have been described in *A. baumannii*, including BfmSR associated with morphology, biofilm formation and adhesion to eukaryotic cells (Tomaras et al., 2008; Liou et al., 2014), PmrAB, modulating lipid A modifications in response to antimicrobial peptides and polymyxin (Adams et al., 2009; Arroyo et al., 2011), BaeSR and AdeRS, which modulate the expression of the AdeABC efflux pump (Marchand et al., 2004; Lin et al., 2014), CheAY regulating the chaperone/usher pili and AbaI quorum sensing (Chen et al., 2017) and GacAS, associated with pathogenesis and host immune evasion (Cerqueira et al., 2014; Bhuiyan et al., 2016).

While all the TCSs play important roles in bacterial homeostasis and physiology, the GacAS system plays a critical role in bacterial pathogenesis and host-pathogen interactions. GacAS is a global regulator, whereby disruption of either *gacA* or *gacS* abolishes the ability of ATCC 17978 to cause lethality in either *Candida albicans* or murine models (Cerqueira et al., 2014). GacS forms the inner membrane sensor kinase, with conserved histidine and aspartic acid residues at H299 and D719/771, respectively, responsible for the phosphorylation of the transcriptional response regulator GacA (Cerqueira et al., 2014). Although GacAS regulates a variety of genes, including *ompA*, *csu* operon, and *motB* to name but a few, its role in the regulation of the phenylacetate catabolite pathway (*paa* operon) is a significant factor contributing to pathogenesis (Cerqueira et al., 2014). Inhibition of this pathway resulted in increased neutrophil migration to the site of infection and bacterial clearance (Cerqueira et al., 2014; Bhuiyan et al., 2016). It should be noted though, consistent with other previously described TCSs, GacAS does display some degree of cross regulation with others; however, *paa* transcriptional regulation is specific to GacAS.

#### Post-transcriptional Regulation by Hfq

The global RNA chaperone Hfq is an important regulator of bacterial virulence in a range of pathogens. In *Salmonella* and *E. coli*, *hfq* mutations result in pleotropic effects, including reduced growth rates, changes in motility, biofilm formation, OMP levels, and attenuated *in vivo* virulence (Tsui et al., 1994; Bossi et al., 2008; Kulesus et al., 2008). Hfq can positively or negatively regulate messenger and/or small RNAs (sRNA) (Jousselin et al., 2009). Interestingly, *Acinetobacter* Hfq is almost double the size of that found in other gamma Proteobacteria, with an elongated C terminal, though the functional significance of this remains unknown (Kuo et al., 2017). Similar to other species, *A. baylyi* and *A. baumannii hfq* mutants display pleotropic phenotypes, including reduced growth rates, elevated sensitivity to environmental stress, reductions in OMV production, fimbriae, biofilm formation and adhesion, invasion and survival in eukaryotic cells (Schilling and Gerischer, 2009; Kuo et al., 2017). Furthermore, changes in cytokine stimulation have been reported with these mutants, though these effects are cell line specific (Kuo et al., 2017). Despite *hfq* not being essential in ATCC 17978, this does not appear to be the case for all strains, as no *hfq* transposon insertion mutant is available in the AB5075 library generated by Gallagher et al. (2015). These datasets highlight the critical need for further investigations into the role of these important and dynamic proteins in different strains in order to elucidate their roles and association with other accessory factors.

## Immune Responses

### Innate Immune Response

#### Cellular Immunity

##### Neutrophils

The importance of neutrophils in response to *A. baumannii* was first noted regarding the increased prevalence of this pathogen in neutropenic patients. However, it was a further 10 years until Faassen and colleagues confirmed their importance in resistance to *Acinetobacter* respiratory infections (Van Faassen et al., 2007). Numerous *in vivo* studies show neutrophil recruitment to the site of infection occurs as early as 4 h, peaking at 24 h, and while their depletion results in enhanced lethality, this effect can be strain specific (Bruhn et al., 2015; Bhuiyan et al., 2016; García-Patiño et al., 2017). The recruitment and activation of neutrophils can be stimulated by a range of host factors, including cytokines and chemokines, though only the bacterial metabolic by-product phenylacetate has been shown to act as a bacterial-derived chemoattractant in the case of *A. baumannii* (Bhuiyan et al., 2016).

Neutrophils elicit antibacterial effects through phagocytosis, degranulation, and neutrophil extracellular trap (NETs) formation (Konstantinidis et al., 2016). Phagocytosis is mediated by TLR activation, IgG opsonization or complement-mediated receptor binding (Nordenfelt and Tapper, 2011). The process is extremely rapid, occurring in as little as 20 s, through pseudopod and filopodia generation, with phagocytic arms capable of retaining and engulfing the bacteria (Nordenfelt and Tapper, 2011; Lázaro-Díez et al., 2017). Once phagocytosed, rapid killing is dependent on ROS and granular fusion, releasing a plethora of antimicrobial molecules, including human defensins and lysosome into the phagosome (Greenwald and Ganz, 1987; Borregaard and Cowland, 1997; Qiu et al., 2009; Nordenfelt and Tapper, 2011; Harding et al., 2017a). However, it is the capacity of neutrophils to kill *A. baumannii* that is contentious, with some *in vitro* studies reporting their co-culture does not affect the viability of either (Kamoshida et al., 2015, 2016). Instead, *A. baumannii* preferentially adhere to the neutrophil surface, in an IL-8-dependent manner, promoting their dissemination (Kamoshida et al., 2016). While others have shown *in vitro* neutrophil phagocytosis kills *A. baumannii*, a finding consistent with *in vivo* studies (Lázaro-Díez et al., 2017). The reasoning behind such conflicting reports is most likely due to the experimental details, in that Kamoshida et al. tested only a single *A. baumannii* strain, ATCC 19606, at 1 h post infection, while Lázaro-Díez et al. performed an extensive assessment, testing 11 strains, including 5 *A. baumannii* and 6 *A. pittii* over a time course, emphasizing the importance of testing multiple strains.

NETs are an important mechanism by which neutrophils control pathogens, though their induction in response to *A. baumannii* is equally controversial (Brinkmann et al., 2004; Brinkmann and Zychlinsky, 2012; Kamoshida et al., 2015, 2018; Konstantinidis et al., 2016). NETs constitute a mesh of chromatin, impregnated with antimicrobial proteins and peptides, including myeloperoxidase, neutrophil elastase, and LL-37, respectively (Konstantinidis et al., 2016). NETs have been linked to the control of bacterial infections, while *A. baumannii* has been reported to inhibit their formation (Kamoshida et al., 2018). This mechanism of inhibition has yet to be fully elucidated and confirmed *in vivo*, though the neutrophil cell surface receptors CD11a and CD11b have been implicated in the observed reduced adhesion of *A. baumannii* to neutrophils (Kamoshida et al., 2018).

While cytokines such as IL-8 and tumor necrosis factor (TNF)-α have stimulatory and chemoattractive effects on neutrophils, it should be noted that TNF-α induces concentration-dependent effects, including cytokine release and MAP kinase activation or cell apoptosis (March et al., 2010; Kikuchi-Ueda et al., 2017). Other host factors such as neutrophil phosphatase, Wip1, and serum amyloid A and P can also regulate neutrophil migration and pro-inflammatory cytokine secretion (Renckens et al., 2006; Sun et al., 2014).

##### Macrophages

Similar to neutrophils, the role of macrophages in *A. baumannii* infection is equally controversial. While their depletion in zebrafish has no effect, in murine models, macrophage depletion reduces pro-inflammatory cytokines and elevates bacterial burdens when depleted in conjunction with complement (Qiu et al., 2012; Bruhn et al., 2015; Bhuiyan et al., 2016). However, macrophages are important early defenders, promoting neutrophil recruitment and phagocytosis (Qiu et al., 2009, 2012). Alveolar macrophages provide the first line of defense against *A. baumannii* in the lungs, capable of microfilament and microtubule-dependent phagocytosis of the bacteria, stimulating high levels of IL-6, TNF-α, and macrophage inflammatory protein-2, potent chemoattractants for neutrophils, with additional IL-1β and IL-10 produced at latter time points (Qiu et al., 2012). Though macrophages kill phagocytosed *A. baumannii,* they do so at a significantly slower rate than that of neutrophils; however, they can phagocytose bacteria in as little as 10 min (Qiu et al., 2012; Lázaro-Díez et al., 2017).

##### Natural Killer Cells, Dendritic Cells, and Mast Cells

Natural killer (NK) cells are an important defense against viral infections, intra- and extracellular bacteria; however, their role in *A. baumannii* infection remains largely unexplored (Small et al., 2008; Waggoner et al., 2016; Paidipally et al., 2018). In a murine pneumonia model, depletion of NK cells led to reduced survival and impaired bacterial clearance, though the impact of the NK cells was indirect, and mediated *via* the production of the neutrophil chemoattractant, keratinocyte chemoattractant (Tsuchiya et al., 2012). *A. baumannii* attachment to the natural cytotoxicity receptors on the surface of NK cells, has very low or no affinity, indicative that the interaction is indirect (Esin et al., 2008). Furthermore, NK cell cytotoxicity is significantly reduced in mice with *A. baumannii* septicaemia compared to uninfected controls; however, the mechanism defining this impact has not been explored (Cirioni et al., 2016).

Dendritic cells (DCs) are unique antigen-presenting cells linking the innate and adaptive immune systems. *A. baumannii* OmpA activates DCs in a dose-dependent manner, resulting in maturation, MAP kinase and NF-ĸB signaling, leading to the induction of a CD4+ Th_1_ T cell responses or early onset apoptosis and delayed necrosis (Lee et al., 2007). DC death is *via* mitochondrial targeting and production of ROS, consistent with OmpAs mechanism of action, suggestive that *A. baumannii* may modulate T cell response *via* this cell type (Lee et al., 2010). Furthermore, despite the identification of neutrophil-DC hybrids several years ago (Geng et al., 2013; Matsushima et al., 2013), it remains to be determined whether they contribute to the control of *Acinetobacter* infections.

Mast cells are considered sentinels of mucosal layers, sensing and responding to pathogen invasion. Lung mast cells have been shown to secrete IL-8 and TNF-α in response to *A. baumannii*, enhancing neutrophil recruitment to the site (Kikuchi-Ueda et al., 2017).

#### Cell Signaling in Response to *Acinetobacter*

##### Toll-Like Receptor Signaling

Toll-like receptor (TLR) signaling is an important mechanism by which hosts recognize and respond to pathogens. Of the 11 reported TLRs, TLR2, 4, and 9 are critical for the recognition and response to *A. baumannii*, through the detection of lipoproteins, peptidoglycan, porins, lipoteichoic acid (TLR2), LOS (TLR4), and unmethylated CpG DNA motifs (TLR9)(Knapp et al., 2006; Lin et al., 2012; Moffatt et al., 2013; Noto et al., 2015).

Host signaling *via* TLR2 and 4 has been extensively documented, whereby activation of either receptor, in the presence or absence of the soluble GPI-linked glycoprotein, CD14, leads to NF-ĸB activation in a MyD88-dependent fashion, resulting in the secretion of pro-inflammatory cytokines including TNF-α; IFN-γ; IL-1, 6, 8, 10, and 12 (Lin et al., 2012; Moffatt et al., 2013; García-Patiño et al., 2017). TLR2 is important for DC recognition of *A. baumannii* OmpA; however, there are conflicting reports regarding the impact of TLR2 knockout, whereby Kim et al. reported increased bacterial burdens in the first 24 h of infection in TLR2 −/− mice, while Knapp et al. observed significantly lower bacterial burdens at the same time point (Knapp et al., 2006; Kim et al., 2014; García-Patiño et al., 2017). These differences may be attributed to the use of different *A. baumannii* isolates, though further investigation is clearly warranted. By contrast, TLR4 −/− and/or CD14 −/− both result in increased bacterial burdens in pneumonia models, as a result of reduced pro-inflammatory cytokine responses and neutrophil recruitment. Interestingly though, TLR4 knockout has also been shown to reduce the lethality of *A. baumannii* septicaemia, by limiting septic shock caused by LOS (Lin et al., 2012). Consistent with this finding, *A. baumannii* virulence has also been linked to the levels of LOS shedding and TLR4 signaling (Lin et al., 2012). Similarly, the treatment of *A. baumannii*-infected animals with LpxC inhibitors protects against lethality, by enhancing TLR2 stimulation and reducing NF-ĸB signaling and TNF-α secretion by four- and two-fold respectively, promoting opsonophagocytic killing in response to increased surface PNAG (Lin et al., 2012; Moffatt et al., 2013). By contrast, *A. baumannii* isolates with phosphoethalomine-modified lipid A induce significantly higher levels of TLR4 signaling compared to unmodified LOS (Lin et al., 2012).

In contrast to TLR2 and TLR4, TLR9 signaling in response to *A. baumannii* is the least well characterized. TLR9 is an internal receptor of the endolysosome, responsible for the detection of bacterial and viral DNA (Noto et al., 2015; Harding et al., 2017a). Similarly, its stimulation promotes NF-ĸB activation and pro-inflammatory cytokine responses, whereby TLR9 −/− mice have reduced TNF-α and IFN-ϒ in response to *A. baumannii* lung infections, causing elevated bacterial burdens, systemic dissemination, and increased tissue damage (Noto et al., 2015).

##### Soluble Secreted Factors

The production of pro-inflammatory cytokines in response to *A. baumannii* is primarily *via* NF-ĸB activation, with each cytokine driving a different response. For example, NLRP3- caspase 1- caspase 11 activation leads to the release of IL-1β and IL-18 from infected lung epithelia resulting in tissue damage (Dikshit et al., 2017). IL-17, however, promotes granulocytopoiesis and drives secretion of GM-CSF and IL-8, stimulating the antimicrobial peptide LL-37 (García-Patiño et al., 2017). With the release of IL-8 and TNF-α, neutrophils are recruited and activated (March et al., 2010; Bist et al., 2014). However, while IL-33 represses IL-8 secretion, it is known to also promote neutrophil migration and stimulation (Peng et al., 2018).

Epithelial cells and neutrophils also secrete an array of antimicrobial peptides, including human β-defensin 2 and 3, the cathelicidin LL-37 and CD14 enhancing TLR4 ligand interactions through myeloid differentiation factor 2 binding (March et al., 2010; García-Patiño et al., 2017; Harding et al., 2017a). *A. baumannii* is highly susceptible to human β-defensin 2, which causes membrane disruption and increased permeability, although the potential for cell death *via* non-membrane lytic methods (i.e., inhibition of nucleic acid synthesis) or synergy with other antimicrobial peptides cannot be excluded (Cobo and Chadee, 2013). Interestingly, only LOS-deficient *A. baumannii* demonstrates elevated sensitivity to the antimicrobial peptide LL-37, despite the target considered to be LOS (Moffatt et al., 2013).

##### Intracellular Responses

Despite *A. baumannii* being considered an extracellular pathogen, *in vitro* evidence exists for their ability to invade lung epithelial cells and macrophages (Choi et al., 2008b; Qiu et al., 2012), though this observation is controversial, and the evidence should be viewed with caution, given the limitations of the studies. In both instances, only a single *A. baumannii* strain was tested, with extracellular bacteria eliminated using gentamicin; however, the authors neglect to provide either the minimum inhibitory concentration for their respective strains or suitable microscopy to support their observations. In contrast to these reports, other studies testing a range of *A. baumannii* strains have been unable to visualize a direct interaction or invasion of the lung epithelial cell line A549, irrespective of MOI or incubation duration (Lázaro-Díez et al., 2016). Furthermore, Qiu et al. observed a reduction in bacterial viability during co-culture with macrophages (Qiu et al., 2012), suggesting that these observations are purely phagocytosis as opposed to active invasion and their significance in the context of *in vivo* infection has yet to be confirmed.

In addition to TLR9, the cytosolic family of NOD-like receptors, including Nod1 and Nod2 associated with pathogen-associated molecular pattern recognition, induce NF-ĸB, but not MAP kinase through interactions with Rip2 (Bist et al., 2014). This NF-ĸB activation induces IL-8, TNF-α and β-defensin, although it should be noted these responses are cell type specific (Bist et al., 2014). ROS and nitric oxide play critical and moderate roles in the control of intracellular *A. baumannii,* respectively (Qiu et al., 2009, 2012). Evident by the fact that gp91^phox^−/− mice, deficient in the NADPH phagosomal oxidase (and thus ROS), are more susceptible to *A. baumannii* infection than neutropenic mice, while those defective for nitric oxide production demonstrate only moderate increases in bacterial burdens (Qiu et al., 2009).

##### Complement-Mediated Killing

Complement-mediated killing is an important non-cellular innate immune component, consisting of multiple soluble factors, acting in a cascade promoting either bacterial cell lysis or opsonophagocytosis. Three pathways exist for the deposition of complement factors on to bacterial surfaces, although in human serum, the alternative complement pathway is required for *A. baumannii* killing (Garcia et al., 2000; Kim et al., 2009; King et al., 2009; Bruhn et al., 2015). Resistance is frequently reported in clinical *A. baumannii* isolates and *in vitro* serum resistance may correlate with more severe disease (Bruhn et al., 2015).

The alternative complement pathway is regulated by factor H, a soluble component important for the recognition of host cell markers (Ferreira et al., 2010). Activation of the alternative complement pathway leads to the deposition of C3 on the surface of serum-sensitive isolates, though discrepancies exist surrounding the binding of factor H to bacterial cells and subsequent inhibition of C3 deposition, promoting serum resistance in *A. baumannii* (Dave et al., 2001; King et al., 2009; Laarman et al., 2011). Previously, Kim et al. reported that factor H bound to *A. baumannii* OMPs, promoting serum resistance (Kim et al., 2009). While King et al. were unable to identify bound factor H on their serum-resistant isolates (King et al., 2009), suggesting that factor H acquisition is not solely responsible.

Furthermore, the *A. baumannii* plasminogen-binding protein, CipA, was also shown to inhibit the alternative complement pathway *via* C3b cleavage and degradation of fibrin networks, by mechanisms that have yet to be fully elucidated (Koenigs et al., 2016). Consistent with the role of CipA in complement resistance, *cipA* deletion mutants are also more susceptible to killing by human serum (King et al., 2013).

Genes involved in *A. baumannii* cell envelope homeostasis also contribute to serum resistance. For example, disruption of the TCS *bfmS*, which regulates pilus and capsule biosynthesis, leads to serum resistance (Geisinger et al., 2018). *A. baumannii* genes associated with capsule biosynthesis and glycosylation, including *ptk, epsA, mltB,* and *pglC,* encoding a putative tyrosine kinase, OM polysaccharide exporter, lytic transglycosylase and glycosyltransferase, respectively, are required for resistance to killing in human ascites fluid and serum, highlighting the importance of capsule in resisting complement (Russo et al., 2010; Lees-Miller et al., 2013; Crépin et al., 2018).

### Adaptive Immune Response

While numerous studies have examined the ability of different bacterial components to induce a range of adaptive immune responses (McConnell and Pachón, 2010; McConnell et al., 2011a,b; García-Quintanilla et al., 2014; Badmasti et al., 2015; Kuolee et al., 2015; Garg et al., 2016; Ainsworth et al., 2017; Bazmara et al., 2017; Pulido et al., 2018; Song et al., 2018), very little work has been done to examine these during the course of infection, due to several confounding factors, including the available animal models, rate of disease progression and severity.

To our knowledge, the importance of Th_1_ vs. Th_2_ or Th_17_ responses has yet to be fully elucidated with regard to *A. baumannii* infection. Despite IL-17 being important for neutrophil recruitment and secretion of β-defensin, deletion of IL-17A in mice has no impact on bacterial burdens (Breslow et al., 2011; Yan et al., 2016). Furthermore, Qui et al. demonstrated that mice which recover from sub-lethal infections do not have increased survival compared to naïve mice when infected with a lethal dose of *A. baumannii* 6 weeks later, despite having significantly higher levels of CD4+ and CD8α+ T cells and CD19+ B lymphocytes (Qiu et al., 2016). This suggests that adaptive immune responses play only a minor role in the resolution of *A. baumannii* infections. By contrast, some studies have shown the induction of antibody subtypes, IgM and IgG (isotypes IgG1 and 2c), and cytokines, IFN-ϒ, IL-4, and IL-17, can promote bacterial killing and improve host survival (McConnell et al., 2011b; Luo et al., 2012; García-Quintanilla et al., 2014). Further work is needed to clarify the significance of different adaptive immune components in the clearance of and resistance to *A. baumannii* infections.

## *In vivo* Models for the Study of *A. baumannii* Host-Pathogen Interactions

### Mammalian Models

Murine models have been the predominant mammalian model used to study *Acinetobacter* infections over the last 30 years, with a small number of studies also utilizing rabbits (Rodríguez-Hernández et al., 2004; Pachón-Ibáñez et al., 2010) and guinea pigs (Bernabeu-Wittel et al., 2005). While initial studies focused on assessing antibiotic efficacy, more recent studies have investigated bacterial pathogenesis, host interactions, immunity and alternative therapeutic treatments (Obana et al., 1985; Joly-Guillou et al., 1997; Ko et al., 2004; Knapp et al., 2006; Jacobs et al., 2010; Cerqueira et al., 2014; Lood et al., 2015; Noto et al., 2015; Sebe et al., 2016; Dikshit et al., 2017; Murray et al., 2017; Peng et al., 2017). A number of models have been developed for skin and soft tissue infection, endocarditis, osteomyelitis, bloodstream infection, and pneumonia, with the latter two being the most commonly used given the frequency and severity of these infections in hospital and community settings (Peleg et al., 2008a; McConnell et al., 2013).

#### Bloodstream Infection

Several bacterial virulence factors have been confirmed using bloodstream infection models, including the acinetobactin iron acquisition system, the universal stress protein A (UspA), and the TCS GacAS (Gaddy et al., 2012; Cerqueira et al., 2014; Elhosseiny et al., 2015). A recent investigation utilized a bacteremia model to determine the global gene expression profile of *A. baumannii* ATCC 17978 during a life-threatening infection, and compared expression profiles between *in vitro* and *in vivo* growth, revealing 886 differentially expressed genes (Murray et al., 2017).

Recently, bloodstream infection models have been expanded to test the efficacy of bacteriophage therapy to treat lethal infections. Lood et al. used purified, recombinant autolysin from a bacteriophage to treat mice infected with a lethal dose of *A. baumannii,* resulting in increased survival in lysin-treated mice (up to 50%) and reduced *A. baumannii* burdens (Lood et al., 2015). Similar results were observed with a more recent study using another bacteriophage lysin (Peng et al., 2017).

#### Pneumonia

Lung infection models were first used in 1997 to evaluate the efficacy of imipenem *in vivo* against acute *A. baumannii* pneumonia (Joly-Guillou et al., 1997). Two lung infection methods are most commonly used to induce pneumonia in mice: intra-tracheal, where the trachea of an anesthetized mouse is cannulated with a blunt-ended needle and the inoculum instilled; or intranasal, where the inoculum is pipetted over the nares of anesthetized mice (Joly-Guillou et al., 1997; Eveillard et al., 2010). Both methods induce inflammatory and histological responses consistent with acute pneumonia and a number of virulence factors have been assessed using these models, including phospholipase D, OmpA, and a heme oxygenase (Choi et al., 2008b; Jacobs et al., 2010; De Léséleuc et al., 2014). Assessment of essential *A. baumannii* genes in the context of pneumonia has also been performed using a transposon mutant library of *A. baumannii* ATCC 17978 and comparing input and output mutant pools after lung infection (Wang et al., 2014). This approach identified 157 genes, including OmpA and several novel virulence factors that are required for a pneumonia infection.

Over the last 15 years, mouse pneumonia models have been increasingly used to understand the host immune response to *A. baumannii* as described above. Furthermore, vaccine efficacy studies have now also been performed using mouse infection models. (McConnell and Pachón, 2010; McConnell et al., 2011a; Luo et al., 2012; Russo et al., 2013; Qiu et al., 2016).

With the rise in studies, it is also now recognized that different strains of *A. baumannii* elicit different immune responses in mice (De Breij et al., 2012; Dikshit et al., 2017). This is an important finding considering that for many years, strains such as ATCC 17978 and 19606 have been used to study *A. baumannii* pathogenesis; however, they are not representative of the dominant clinical strains found in hospitals today. When compared to international clonal Type I and II strains, they have reduced virulence and elicit different immune responses (Eveillard et al., 2010; De Breij et al., 2012; Dikshit et al., 2017).

### Other Infection Models

As previously described, a number of other infection models exist for studying *A. baumannii* infections. Skin and soft tissue models have been developed in both mice and rats for treatment studies and as a screening tool for determining gene essentiality (Shankar et al., 2007; Russo et al., 2008; Dai et al., 2009). Several groups have tried to develop an osteomyelitis model in mice and rats with varying levels of success (Crane et al., 2009; Collinet-Adler et al., 2011). Crane et al. were able to establish a non-lethal infection in mice by implanting stainless steel pins into their tibias, while Collinet-Adler et al. were unable to establish chronic bone infections in rats (Crane et al., 2009; Collinet-Adler et al., 2011). Rabbits have been used for studying meningitis and endocarditis, and guinea pigs used to study pneumonia (Rodríguez-Hernández et al., 2004; Bernabeu-Wittel et al., 2005; Pachón-Ibáñez et al., 2010). While larger animals allow researchers to sample the same animal at multiple time points, the increased costs and ethical concerns associated with larger animals have limited their use thus far.

The increasing costs and growing ethical concerns regarding mammalian models have spurred the development of non-mammalian models such as *C. elegans*, *G. mellonella*, and zebrafish for studying *A. baumannii* host-pathogen interactions. Despite their clear differences from mice and humans, non-mammalian models have proved useful for the assessment of mutants, screening compounds, and enabling visualization of host-pathogen interactions.

#### Caenorhabditis elegans

*C. elegans*, a small soil-dwelling nematode was the first non-mammalian model to be used to study *A. baumannii* pathogenesis (Smith et al., 2004). The small size (1 mm), transparency, short replication cycle (2–3 days), and well-characterized genome make it an ideal model to study bacterial-host interactions. It was first used by Smith et al. to study *A. baumannii* pathogenesis in the presence of ethanol, with a later method developed using proliferation and brood size as the outcome measure rather than worm death (Smith et al., 2004, 2007). Polymicrobial interactions between *A. baumannii* and *Candida albicans* have also been assessed in *C. elegans,* where *A. baumannii* reduced *C. albicans* filament production and virulence within the worm (Peleg et al., 2008b). *C. elegans* has most recently been used to screen potential antimicrobial agents (Jayamani et al., 2015; Mohamed et al., 2017). Jayamani et al. used a liquid infection assay in a 384-well format to concurrently assess the antimicrobial activity of peptides in parallel to evaluating host toxicity, revealing 15 molecules that prolonged worm survival (Jayamani et al., 2015). A disadvantage of *C. elegans* though is their requirement to be maintained at 25°C, with 37°C causing worm death. This may impact the virulence of pathogens including *A. baumannii* (Peleg et al., 2009; De Silva et al., 2017).

#### Galleria mellonella

*G. mellonella* (caterpillars of the greater wax moth) have a considerable advantage over *C. elegans* in that they can be maintained at 37°C and a precise inoculum or therapeutic dose can be administered into the body of the caterpillar (Peleg et al., 2009; Hornsey et al., 2013). *G. mellonella* also have a more advanced immune system with both humoral and phagocytic cells (Peleg et al., 2009). *A. baumannii* kills *G. mellonella* in a dose-dependent manner and survival can be prolonged by the administration of antibiotics (Peleg et al., 2009). *G. mellonella* has since been used to test the efficacy of antibiotic combinations, assess the virulence of mutants, or compare pathogenicity between strains (Peleg et al., 2009; Antunes et al., 2011; Hornsey and Wareham, 2011; Gaddy et al., 2012; Iwashkiw et al., 2012; Wand et al., 2012; Hornsey et al., 2013; O’Hara et al., 2013; Yang et al., 2015; Betts et al., 2017). A disadvantage of note is that *G. mellonella* sourced from different suppliers can show significant variance in results (Betts et al., 2017). However, with their increasing use, more standardized models have been developed that control for age, size, and food supply.

#### Dictyostelium discoideum

*D. discoideum* is a unicellular amoeba that feeds on bacteria, with previous work having highlighted similarities between amoeba phagocytosis and mammalian immune cell phagocytosis (Hasselbring et al., 2011). It has therefore been proposed that when used in conjunction with other non-mammalian models, such as *C. elegans,* which kill *via* extracellular methods, the two can represent multiple areas of a mammalian infection (Smith et al., 2007). In order to determine whether bacteria are killed by *D. discoideum,* liquid cultures of the two are mixed and plated, allowing the faster growing bacteria to form a lawn, with formation of plaques if amoeba are able to phagocytose bacteria (Smith et al., 2007; Iwashkiw et al., 2012; Weber et al., 2013). *D. discoideum* and *C. elegans* have been jointly used to screen transposon mutant libraries for attenuated mutants (Smith et al., 2007). Two other studies were performed using *D. discoideum* and *C. elegans* and showed that the T6SS is not required for virulence, while a glycosylation pathway was (Iwashkiw et al., 2012; Weber et al., 2013).

#### Zebrafish

Zebrafish (*Danio rerio*) are the most recent non-mammalian model to be utilized for host-pathogen interaction studies. Zebrafish are a small (3–4 cm) freshwater fish native to India, Pakistan, and Bhutan and are susceptible to a range of bacteria, including *Vibrio cholera, S. aureus*, and *Shigella flexneri* (Prajsnar et al., 2008; Mostowy et al., 2013; Runft et al., 2014). A considerable advantage of zebrafish to other non-mammalian models is their advanced immune system development with innate immune cells present at 25 h post fertilization and a fully functional adaptive immune system developed by adulthood (Van Der Sar et al., 2004). Transparency can be maintained to allow for real-time visualization, numerous transgenic fish lines are available, including fluorescently tagged neutrophils and macrophages and immune cell-depleted fish (Hall et al., 2007; White et al., 2008; Ellett et al., 2010; Li et al., 2010). The establishment of zebrafish to study *A. baumannii* pathogenesis has recently been published, and showed that *A. baumannii* is lethal toward zebrafish in a dose-dependent manner, with interactions between *A. baumannii* and neutrophils easily imaged (Bhuiyan et al., 2016). The unique advantages of real-time imaging during infection allowed the authors to elucidate novel findings related to neutrophil migration in the context of an *A. baumannii* infection.

## Conclusions

Taken together, this review highlights the real advances that have been made in our understanding of *A. baumannii* pathogenesis, but further highlights areas in need of more in-depth analysis. Consistency and transparency in the field and subsequent publications will optimize the success of future studies. The selection of multiple and appropriate *A. baumannii* strains, encompassing those that are representative of modern-day clinical isolates and human infections, combined with the standardization of cell lines, animal models, and procedures will provide a level of uniformity across all studies. Such consistency will not only enable direct comparison between studies, but also advance our overall understanding of this important pathogen.

However, the future does hold promise with more innovative techniques and the rapid advancement of technology, new approaches are providing greater insight into the intricacies of regulatory networks not only in the bacterial cell but also in the host. As cross-disciplinary research continues to grow, so too does our understanding of this important pathogen and its hosts.

## Author Contributions

CD wrote the “*In vivo models*” section and the “Introduction”. XK wrote the “Natural Killer Cells, Dendritic Cells, and Mast Cells” section. MU wrote the section “Neutrophils”. FM wrote all remaining sections and prepared the figures. AP edited the manuscript.

### Conflict of Interest Statement

The authors declare that the research was conducted in the absence of any commercial or financial relationships that could be construed as a potential conflict of interest.
